# Clinical Significance of Extramural Tumor Deposits in the Lateral Pelvic Lymph Node Area in Low Rectal Cancer: A Retrospective Study at Two Institutions

**DOI:** 10.1245/s10434-016-5379-9

**Published:** 2016-07-08

**Authors:** Ryoma Yagi, Yoshifumi Shimada, Hitoshi Kameyama, Yosuke Tajima, Takuma Okamura, Jun Sakata, Takashi Kobayashi, Shin-ichi Kosugi, Toshifumi Wakai, Hitoshi Nogami, Satoshi Maruyama, Yasumasa Takii, Takashi Kawasaki, Kei-ichi Honma

**Affiliations:** 1 Division of Digestive and General Surgery, Niigata University Graduate School of Medical and Dental Sciences, Niigata, Japan; 2 Department of Digestive and General Surgery, Uonuma Institute of Community Medicine, Niigata University Medical and Dental Hospital, Niigata, Japan; 3 Department of Surgery, Niigata Cancer Center Hospital, Niigata, Japan; 4 Department of Pathology, Niigata Cancer Center Hospital, Niigata, Japan

## Abstract

**Background:**

The presence of extramural tumor deposits without lymph node structure (EX) is an important prognostic factor for patients with colorectal cancer. However, the clinical significance of EX in the lateral pelvic lymph node area (LP-EX) remains unclear. This study aimed to determine the prognostic implications of LP-EX for patients with low rectal cancer.

**Methods:**

This retrospective study involved 172 consecutive patients with stage 2 or 3 low rectal cancer who underwent curative surgery including lateral pelvic lymph node (LPLN) dissection. The patients were classified into the following three groups according to the metastatic status of the LPLN area: patients without metastasis (no-LP-M group), patients with lymph node metastasis (LP-LNM group), and patients with EX (LP-EX group). Potential prognostic factors of overall survival (OS) and relapse-free survival (RFS) were identified in uni- and multivariate analyses.

**Results:**

Classification assigned 131 patients (76 %) to the no-LP-M group, 27 patients (16 %) to the LP-LNM group, and 14 patients (8 %) to the LP-EX group. The 5-year OS rate was 80.3 % in the no-LP-M group, 61.1 % in the LP-LNM group, and 34.9 % in the LP-EX group (*P* < 0.001). The corresponding 5-year RFS rates were 62.2, 33.8, and 14.3 %, respectively (*P* < 0.001). A multivariate Cox proportional hazards regression analysis showed that the presence of LP-EX was an independent prognostic factor for OS (*P* = 0.006) and RFS (*P* = 0.001).

**Conclusions:**

The LP-EX classification is a useful pathologic parameter that can be used to stratify patients with metastasis in the LPLN area.

Lateral pelvic lymph node (LPLN) metastasis of low rectal cancer is managed quite differently between Western countries and Japan. Western countries generally consider LPLN metastasis to be a systemic disease, and neoadjuvant chemoradiotherapy (NACRT) followed by total mesorectal excision (TME) is the standard treatment for low rectal cancer.^1^^–^3 On the other hand, in Japan, LPLN metastasis is regarded as a local disease, and TME with LPLN dissection has been performed for patients with locally advanced low rectal cancer.^4^^,^^5^ Large-scale retrospective studies in Japan have evaluated the survival outcomes for patients with LPLN metastasis, concluding that LPLN metastasis could be regarded as a form of regional lymph node metastasis in low rectal cancer.^6^

Previous reports have demonstrated that the incidence of LPLN metastasis is 10 to 25 % among patients with low rectal cancer 2^,^^7^^,^^8^ and that the presence of LPLN metastasis is a poor prognostic factor.^5^^,^^6^^,^^9^ A retrospective Japanese study of low rectal cancer showed that the overall survival (OS) rate for patients with LPLN metastasis was significantly lower than for patients with mesorectal lymph node metastasis.^6^ Hence, patients with LPLN metastasis should be treated in a manner independent of that for patients who have only mesorectal lymph node metastasis. Moreover, it also may be useful to stratify LPLN metastases into several subclassifications, which could provide further useful information for tailor-made treatments.

Extramural tumor deposits without lymph node structure (EX) have been investigated in studies of colorectal cancer for two decades.^10^^–^17 The categorization of EX by the American Joint Committee on Cancer (AJCC) has changed several times. The AJCC 5th edition^18^ proposed the first categorization of EX, determined on the basis of size. An EX with a diameter greater than 3 mm was classified in the N category as a lymph node metastasis, whereas an EX with a diameter up to but not exceeding 3 mm was classified in the T category as a discontinuous tumor extension. In contrast, the criteria for EX categorization relied on contour in the AJCC 6th edition,^19^ which recommended that a tumor nodule be classified in the N category if the nodule had a smooth contour and in the T category if the nodule had an irregular contour.

Currently, the categorizations of EX according to size and contour have been abandoned. Instead, the AJCC 7th edition^20^ notes that a peritumoral deposit or satellite nodule in the pericolic or perirectal fat (which may represent discontinuous spread, extravascular spread, or a totally replaced lymph node) is recorded as a tumor deposit (TD). Regarding the treatment of TD in the tumor-node-metastasis (TNM) classification, the AJCC 7th edition states that totally replaced nodes should be counted separately as positive nodes in the N category, whereas discontinuous spread or venous invasion should be classified and counted in the site-specific factor category. However, the AJCC 7th edition does not comment on the site of EX. It has been unclear whether TD should be categorized for all regional lymph node areas, including the LPLN area.

In the current study, we clarified the clinical significance of EX in the LPLN area (LP-EX) and elucidated the optimal categorization of LP-EX in patients with low rectal cancer. We enrolled 172 consecutive patients who had undergone LPLN dissection with TME and analyzed their clinicopathologic characteristics with respect to survival and recurrence outcomes.

## Materials and Methods

### Patients

This retrospective study involved 172 consecutive patients with stage 2 or 3 low rectal cancer who underwent R_0_ resection that included both TME and LPLN dissection at Niigata University Medical and Dental Hospital or Niigata Cancer Center Hospital between January 2000 and December 2012. Patients with histologically confirmed adenocarcinoma were selected from our colorectal databases according to the AJCC 7th edition.^10^

In the current study, low rectal cancer was defined as tumor with the distal edge located at or below the peritoneal reflection. We did not apply NACRT at our institutions during the study period because it remains controversial whether this approach improves OS or contributes to the benefits of sphincter-preserving surgery.^21^ After TME with LPLN dissection, the patients were followed up by physical and laboratory testing, as well as by imaging. Recurrences were classified as local or distant according to the Japanese Society for Cancer of the Colon and Rectum (JSCCR) classification.^22^ Local recurrence was defined as any tumor recurrence within the true pelvis or anal canal. Distant recurrence was defined as any tumor recurrence outside the pelvis. Carcinoembryonic antigen and carbohydrate antigen 19–9 were monitored periodically. Disease recurrence was mainly determined by chest-abdominal-pelvic computed tomography scans. Colonoscopy was performed to detect local recurrence at the anastomotic site.

The current study included 122 men and 50 women with a median age of 63 years (range, 17–81 years). Of these 172 patients, 103 received postoperative adjuvant chemotherapy, with 97 receiving 5-fluorouracil (5-FU)- based adjuvant chemotherapy (5-FU, 5-FU/leucovorin, 5-FU/mitomycin C, tegafur-uracil/leucovorin, tegafur-gimeracil-oteracil potassium capsule, doxifluridine, or 1-hexylcarbamoyl-5-fluorouracil) and 6 receiving oxaliplatin-based adjuvant chemotherapy (5-FU/leucovorin plus oxaliplatin [FOLFOX] or capecitabine plus oxaliplatin [CapeOX]). On the other hand, 69 patients received no adjuvant chemotherapy. The median follow-up period for the 172 patients was 49.5 months (range, 0.4–168.9 months) (Table 1). This study was approved by the institutional review board at each institution.

**Table 1:** Association between the status of the LPLN area and other clinicopathologic variables

Variable	No-LP-M	LP-LNM	LP-EX	*P* value
	(*n* = 131)	(*n* = 27)	(*n* = 14)	
Age (years)				
<65	76	14	6	0.501
≥65	55	13	8	
Sex				
Male	94	21	7	0.163
Female	37	6	7	
Tumor size (mm)				
<60	73	18	6	0.329
≥60	58	9	8	
T category				
T_2_, T_3_	117	23	12	0.788
T_4_	14	4	2	
Histopathologic grade				
G_1_	22	1	0	0.059
G_2_, G_3_	109	26	14	
Lymphatic invasion				
Absence	46	4	1	0.017
Presence	85	23	13	
Venous invasion				
Absence	44	6	0	0.022
Presence	87	21	14	
N category				
N_0_	52	0	0	<0.001
N_1_, N_2_	79	27	14	

*LP-M* lateral pelvic-metastasis, *LP-LNM* lateral pelvic-lymph node metastasis, *LP-EX* lateral pelvic-extramural tumor deposits

### Anatomy of the LPLN Area and Indication for LPLN Dissection

**Fig. 1:**
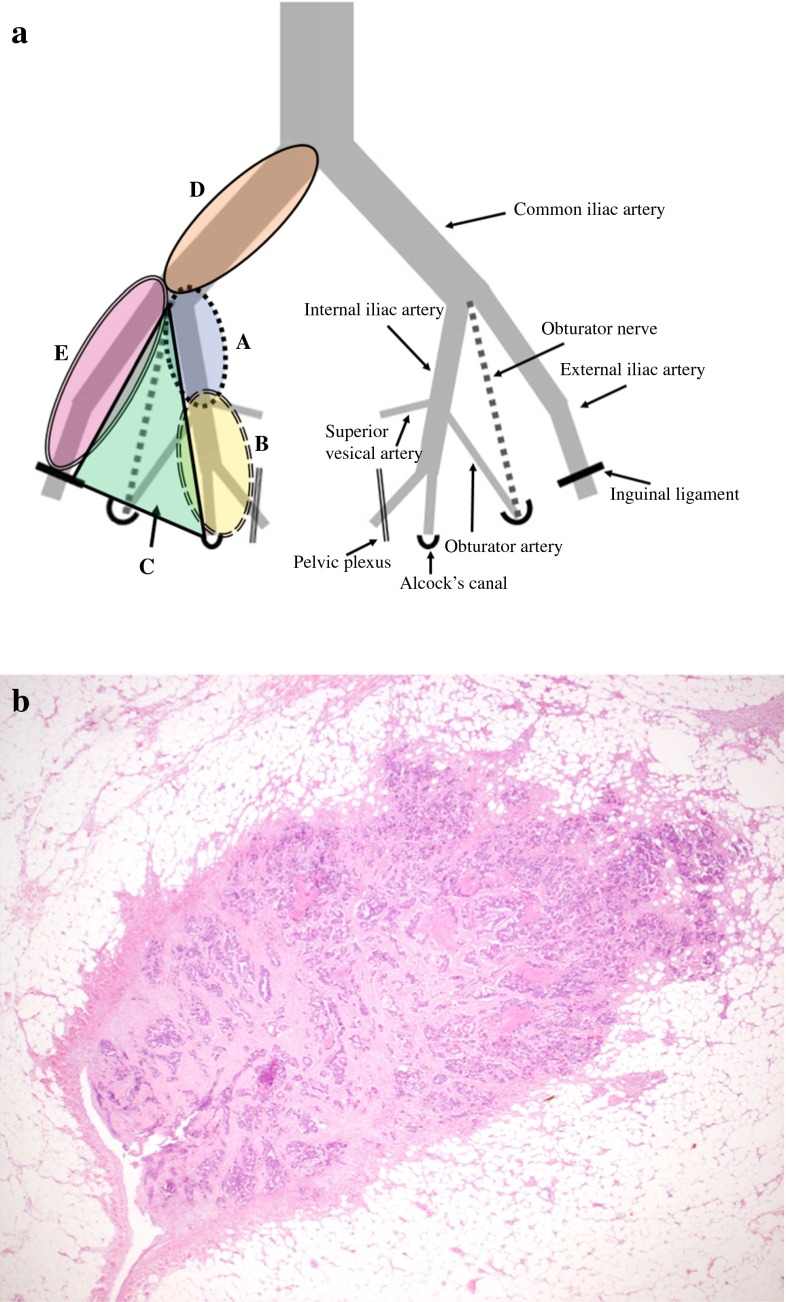
Lymph nodes of the lateral pelvic area. **a** Extramural tumor deposit without lymph node structure (hematoxylin and eosin; ×1). **b**. *A*, *B*, *C*, *D*, and *E* denote proximal internal iliac nodes, distal internal iliac nodes, obturator nodes, common iliac nodes, and external iliac nodes, respectively

The LPLN area was divided into the following five areas according to the JSCCR classification: proximal internal iliac, distal internal iliac, obturator, common iliac, and external iliac (Fig. 1a).^22^ The indication for LPLN dissection followed the JSCCR Guidelines. The lower border of the tumor was located distal to the peritoneal reflection, and the tumor invaded beyond the muscularis propria.^23^ After TME, bilateral LPLN dissection was performed in accordance with previously reported methods.^4^^,^^5^

### Pathologic Examination of LP-EX

After the surgery, surgeons harvested both mesenteric lymph nodes and LPLNs from fresh surgical specimens. Thereafter, the bowel specimen, mesenteric lymph nodes, and LPLNs were fixed in 10 % formalin and sent to the pathology department for routine pathologic examination.

In the current study, LP-EX was defined as a tumor nodule in the LPLN area without histopathologic evidence of residual lymph node structure (Fig. 1b). The LP-EX classification included venous invasion with extravascular spread, a totally replaced node, and discontinuous cancer spread of unknown origin in the LPLN area. One of the authors (Yoshifumi Shimada) retrospectively reexamined all slides of harvested LPLNs and subclassified the metastases in the LPLN area (LP-M) into “lymph node metastasis” (LP-LNM) or “EX” (LP-EX).

### Definitions of the No-LP-M, LP-LNM, and LP-EX Groups

The 172 patients in the current study were classified into three groups according to the pathologic status of the LPLN area. The patients without metastasis in the LPLN area were defined as the “no lateral pelvic metastasis” (no-LP-M) group. The patients with metastasis in the LPLN area were subclassified into two groups (LP-LNM and LP-EX groups). The LP-LNM group comprised patients who had LP-LNM without LP-EX, whereas the LP-EX group consisted of patients who had LP-EX with or without LP-LNM. In other words, the definition of the LP-EX group included patients with LP-EX alone as well as patients with both LP-LNM and LP-EX.

### Prognostic Factors

To elucidate the factors influencing OS and relapse-free survival (RFS) after TME with LPLN dissection, the following nine clinicopathologic variables were tested in all 172 patients: age (<65 vs ≥65 years), sex, tumor size (<60 vs ≥60 mm), T category (T_2_ and T_3_ vs T_4_), histopathologic grade (G_1_ vs G_2_ and G_3_), lymphatic invasion (absence vs presence), venous invasion (absence vs presence), N category (N_0_ vs N_1_ and N_2_), and LP-M (no-LP-M vs LP-LNM vs LP-EX).

### Statistical Analyses

Statistical analyses were performed with IBM SPSS Statistics 22 (IBM Japan Inc., Tokyo, Japan). The relationships between each of the clinicopathologic variables and the pathologic status of the LPLN area were analyzed using the Chi square test. The 5-year OS, RFS, and cumulative local recurrence rates were estimated using the Kaplan–Meier method. The log-rank test was used to assess the statistical significance of differences between subgroups in univariate analyses. Factors with *P* values <0.05 in the univariate analyses were entered into the multivariate analyses. In the multivariate analyses, the Cox proportional hazards regression model was used to identify factors independently associated with OS and RFS after surgery. All *P* values <0.05 were considered statistically significant.

## Results

### Anatomic Sites of LP-LNM

In 54 nodes found in 32 (18.6 %) of the 172 patients, LP-LNM was observed. These 32 patients were assigned to the LP-LNM group or the LP-EX group. The 27 patients with LP-LNM alone were assigned to the LP-LNM group, and the five patients with both LP-LNM and LP-EX were assigned to the LP-EX group. The sites and frequencies of LP-LNM were as follows: proximal internal iliac nodes (2 nodes), distal internal iliac nodes (35 nodes), obturator nodes (12 nodes), common iliac nodes (5 nodes), and external iliac nodes (0 nodes).

### Anatomic Sites of LP-EX

In 14 (8.1 %) of the 172 patients, 23 foci of LP-EX were observed. Each of these 14 patients was assigned to the LP-EX group. The sites and frequencies of LP-EX were as follows: proximal internal iliac nodes (1 focus), distal internal iliac nodes (14 foci), obturator nodes (6 foci), common iliac nodes (2 foci), and external iliac nodes (0 foci).

### Association Between the Pathologic Status of the LPLN Area and Other Clinicopathologic Variables

The status of the LPLN area was significantly associated with lymphatic invasion (*P* = 0.017), venous invasion (*P* = 0.022), and N category (*P* < 0.001), whereas no significant associations were observed between the status of the LPLN area and the other clinicopathologic variables (Table 1).

### Factors Influencing OS and RFS

**Fig. 2:**
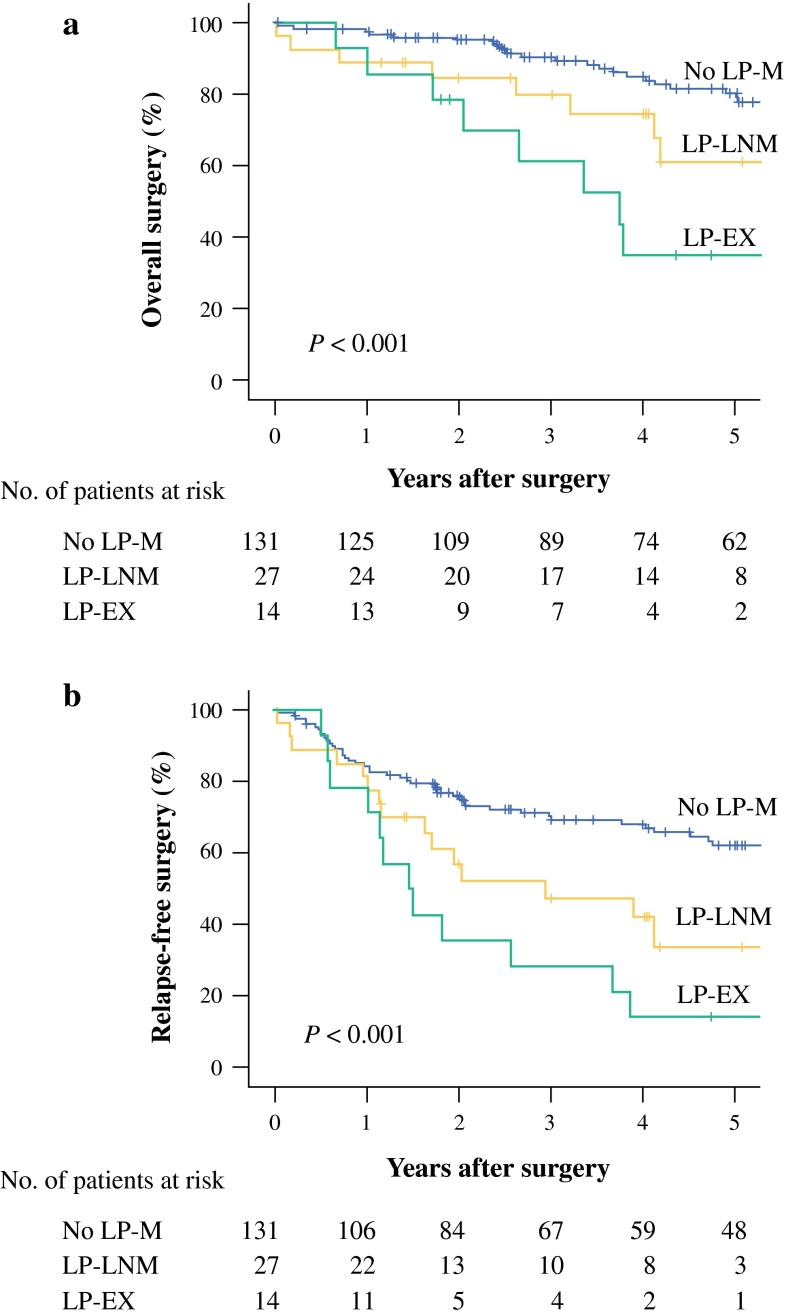
Overall survival curves according to the status of lateral pelvic metastasis. **a** Relapse-free survival curves according to the status of lateral pelvic metastasis. **b** No lateral pelvic-metastasis (no-LP-M), lateral pelvic-lymph node metastasis (LP-LNM), and lateral pelvic-extramural tumor deposit (LP-EX)

The 5-year OS rates after TME with LPLN dissection were 80.3 % in the no-LP-M group, 61.1 % in the LP-LNM group, and 34.9 % in the LP-EX group (Fig. 2a). The univariate analyses showed that N category and LP-M were significant prognostic factors for OS (*P* = 0.006 and <0.001, respectively). These significant variables were entered into a multivariate analysis, which identified LP-EX as a significant independent prognostic factor for OS (*P* = 0.006) (Table 2).

**Table 2:** Uni- and multivariate analyses of different prognostic factors for overall survival and relapse-free survival

Variable	Method	*n*	Univariate		Multivariate		Univariate		Multivariate	
			5-year OS (%)	*P* value	HR (95 % CI)	*P* value	5-year RFS (%)	*P* value	HR (95 % CI)	*P* value
Age (years)	<65	96	77.9	0.231			55.1	0.800		
	≥65	76	67.8				50.8			
Sex	Male	122	73.7	0.529			56.5	0.620		
	Female	50	72.7				46.5			
Tumor size (mm)	<60	97	76.6	0.431			52.2	0.773		
	≥60	75	69.7				54.6			
T category	T_2_, T_3_	152	73.7	0.531			56.4	0.009	1.00	
	T_4_	20	71.5				30.5		2.22 (1.21–4.07)	0.010
Histopathologic grade	G_1_	23	84.4	0.127			64.4	0.217		
	G_2_, G_3_	149	71.7				52.4			
Lymphatic invasion	Absence	51	82.5	0.223			51.9	0.883		
	Presence	121	70.1				53.8			
Venous invasion	Absence	50	75.7	0.871			51.4	0.868		
	Presence	122	72.8				54.2			
N category	N_0_	52	89.2	0.006	1.00		65.8	0.015	1.00	
	N_1_, N_2_	120	67.1		2.36 (0.95–5.85)	0.064	47.7		1.52 (0.82–2.82)	0.184
LP-M	No-LP-M	131	80.3	<0.001	1.00		62.2	<0.001	1.00	
	LP-LNM	27	61.1		1.77 (0.83–3.76)	0.141	33.8		1.67 (0.89–3.11)	0.108
	LP-EX	14	34.9		3.16 (1.39–7.17)	0.006	14.3		3.05 (1.55–5.98)	0.001

*OS* overall survival, *HR* hazard ratio, *CI* confidence interval, *RFS* relapse-free survival, *LP-M* lateral pelvic-metastasis, *LP-LNM* lateral pelvic-lymph node metastasis, *LP-EX* lateral pelvic-extramural tumor deposit

The 5-year RFS rates after TME with LPLN dissection were 62.2 % in the no-LP-M group, 33.8 % in the LP-LNM group, and 14.3 % in the LP-EX group (Fig. 2b). The univariate analyses showed that T category, N category, and LP-M were significant prognostic factors for RFS (*P* = 0.009, 0.015, <0.001, respectively). These significant variables were entered into a multivariate analysis, which identified T category and LP-EX as significant independent prognostic factors for RFS (*P* = 0.010 and 0.001, respectively) (Table 2).

### Cumulative Local Recurrence

The 5-year cumulative local recurrence rates after TME with LPLN dissection were 9.2 % in the no-LP-M group, 26.8 % in the LP-LNM group, and 28.6 % in the LP-EX group. The cumulative local recurrence rate in the LP-EX group was significantly greater than in the no-LP-M group (*P* = 0.009), whereas no significant difference was observed between the LP-LNM and LP-EX groups.

### Pattern of Initial Recurrence in the LP-EX Group

Initial distant recurrences were significantly more likely in the LP-EX group (9/14, 64.3 %) than in the other groups (42/158, 26.6 %) (*P* = 0.006). In contrast, the rate of initial local recurrences did not differ significantly between the LP-EX group (3/14, 21.4 %) and the other groups (15/158, 9.5 %) (*P* = 0.167).

## Discussion

The current study showed two main results concerning LP-EX in patients with low rectal cancer. First, metastases in the LPLN area could be subclassified as LP-LNM or LP-EX, which were associated with significantly different survival rates. Second, the multivariate analyses showed that LP-EX was an independent prognostic factor. These results indicate that patients with metastasis in the LPLN area could be stratified in a simple manner according to the morphologic evaluation of metastasis in the LPLN area and that the presence of LP-EX might have an important role in tailor-made treatment strategies.

The importance of lateral spreading in low rectal cancer was first brought to the attention of the medical community by Sauer and Bacon^24^ in 1951. In more recent years, several studies have reported the clinical significance of LPLN metastasis and have attempted to stratify patients with LPLN metastasis.^5^^,^^6^^,^^9^^,^^25^^–^28 Ueno et al.^27^ demonstrated that the number of LPLN metastases had a significant association with prognosis: patients with two or more LPLN metastases demonstrated a poorer prognosis than those with only one LPLN metastasis. Akiyoshi et al.^6^ stratified patients with LPLN metastasis according to anatomic location, finding that patients with external LPLN metastasis showed a worse prognosis than those with internal LPLN metastasis. Komori et al.^28^ showed that extracapsular invasion in the LPLN area was an independent prognosis factor correlated with OS and RFS rates. However, to date, no studies have addressed the clinical significance of EX in the LPLN area. Hence, the current study is the first to elucidate the clinical significance of LP-EX.

To date, it has been unclear whether LP-LNM and LP-EX have the same prognostic values. The current study evaluated the prognostic value of LP-EX by comparing it with that of LP-LNM and demonstrated that the 5-year OS and RFS rates for patients with LP-EX (34.9 and 14.3 %, respectively) were considerably worse than for patients with LP-LNM (61.1 and 33.8 %, respectively). Furthermore, we found that the 5-year OS rate for the patients with LP-EX (34.9 %) resembled that for the patients with stage 3C disease (33.4 %), as demonstrated in the AJCC 7th edition.^10^ We have provided the first clarification of the clinical significance of LP-EX, which is sometimes observed in cases of low rectal cancer.

The current study showed that both the OS and RFS rates were significantly worse in the LP-EX group than in the other groups. Moreover, distant recurrences were significantly more common in the LP-EX group than in the other groups, although no significant difference in local recurrences was observed between the LP-EX group and the other groups. The high incidence of distant recurrence may be one of the reasons why the LP-EX group had a poor prognosis, indicating that LP-EX might be a systemic disease rather than a local disease. We consider aggressive adjuvant chemotherapy to be essential for patients with LP-EX and recommend that oxaliplatin-based adjuvant chemotherapy such as FOLFOX or CapeOX should be applied for these patients to improve their oncologic outcome.^1^

This study had three main limitations. First, this was a retrospective study conducted at two institutions and thus was subject to various biases. Second, the incidence of LPLN metastasis might have been underestimated in the current study because we examined only the harvested LPLN tissue rather than all the tissue from the LPLN area. Third, this study did not include patients who underwent NACRT, which is regarded as the standard treatment in Western countries. Additional research is needed to explore LP-EX from other perspectives, for example, in terms of its clinical significance after NACRT.

In conclusion, LP-EX is a useful pathologic parameter that can be used to stratify patients with metastasis in the LPLN area.
